# The habitual characteristic of smart phone use under relevant cues among Chinese college students

**DOI:** 10.3389/fpsyg.2023.1218886

**Published:** 2023-09-20

**Authors:** Ming Li, Jieyue Duan, Yuning Liu, Jingxin Zou, Xuesong Yang, Hong Zeng

**Affiliations:** ^1^Department of Psychology, Guangzhou University, Guangzhou, China; ^2^College of Science and Engineering, Jinan University, Guangzhou, China; ^3^Zhuhai Center for Chronic Disease Control, Zhuhai, China; ^4^School of Philosophy, Psychology and Language sciences, University of Edinburgh, Edinburgh, United Kingdom; ^5^International Joint Laboratory for Embryonic Development and Prenatal Medicine, Medical College, Jinan University, Guangzhou, China; ^6^Clinical and Counseling Psychology Research Center, Guangzhou University, Guangzhou, China

**Keywords:** habitual smartphone use, problematic smartphone use, cue induced behavior, self-control, HSUS

## Abstract

Excessive smartphone use may be habitual behavior induced by cues associated with the phone. Habitual behavior occurs outside of awareness and is characterized by lack of control. It is not like problematic smartphone use (PSU) (Brand et al., 2016), which is used to either limit behavior or produce pleasure and relieve feelings of pain, stress, and failure despite significant harmful consequences. 62 college students participated in experiments to test the effects of visual cues and self-control, which are the important characteristic of habitual behavior in smartphone-related behavior. The results showed that a significantly larger amount of cue-related phone use behavior occurred in the setting where participants (a) had their smartphones in sight and (b) were given no instructions to exert self-control, compared to when neither of the two conditions was imposed. The habitual model is a useful framework for understanding PSU and can help people avoid it with less stress. The results provide substantial implications for reducing the frequency and duration of smartphone use among college populations.

## 1. Introduction

Smartphones provide convenience and efficiency, and at times are seen as an indispensable aspect of people’s professional and personal lives today (Zangbar et al., 2014). However, there is concern about young adults who spend extended periods of time every day using their smartphones (Lee et al., 2014). One study found that 80% of college students use smartphones for more than 4 h a day, while 44% of this population use smartphones for more than 8 h a day (Duan et al., 2021). This behavior increases the risk of problematic smartphone use (PSU) variety of dysfunctional manifestations (Rosen et al., 2013), where individuals become overly immersed in the virtual world of their smartphones, including excessive or uncontrollable use, preoccupation (Panova and Carbonell, 2018), and neglect of other activities (Fischer-Grote et al., 2019; Sohn et al., 2019), leading to serious consequences including sleep disturbances or depression (Thomée et al., 2011). In recent years, more and more young people were suspected to have PSU, however, can excessive smartphone use really be classified as PSU, or could it be categorized as a type of habitual behavior?

Habitual behavior is defined as an automatic behavior triggered by both external cues (e.g., sound, objects, places, people, and preceding actions) and internal cues (e.g., emotional state, urge) (Verplanken and Melkevik, 2008; Kurz et al., 2015). There is overlap in the characteristics of PSU and habitual smartphone use, with habitual smartphone use appearing to be an important contributor to PSU (Shaffer, 1996; Huisman et al., 2000; Van Deursen et al., 2015). However, unlike PSU, habitual behavior does not necessarily lead to negative consequences, nor does it necessarily produce pleasure or relieve pain or stress. Furthermore, although habitual behavior is learned outside of one’s awareness and may be experienced as uncontrollable, the individual may be aware of the behavior once it has begun or after it has stopped. Once the person is aware of the habitual behavior, it can be changed or less easily induced (Hall and McDonnell, 1999).

In substance addiction research, it was found that exposure to substance-associated cues, instigates physiological, behavioral and subjective reactions. This is a phenomenon of cue-induced reactivity, including craving (craving can be triggered by cue-reactivity) and automatic substance using behavior (Zeng et al., 2018), in which behavior becomes autonomous and can be performed with little attention, intention, or cognitive effort, constituting a “habit” (Knowlton, 2013). For smart phone use behavior, there is some similarity with substance use.

In daily life, when people use smart phone, whether it is for internet gaming, watching micro-video, social networking, or visiting shopping sites, it normally brings people a sense of pleasure and help them escape from bad emotion. During this process, the smartphone itself or smartphone-related clues, such as notification ringtones, which is often associated with the feeling of pleasure, then become the stimulus clues that trigger the use of smart phone (Tiffany et al., 2000). Once the individual is exposed to these stimulus clues, it would trigger the use of smart phone use, forming cue induced reactivity or stimulus–response behavior, overall forming a conditioning processes (Carter and Tiffany, 1999).

Therefore, cue reactivity is developed on the basis of associative learning mechanism (Carter and Tiffany, 1999; Loeber and Duka, 2009), which is mostly unconscious, similar to the characteristic of habitual behavior. When people are under the stimulation of phone cues, they might then increase the average frequencies and extend the durations of their smartphone. In this way, although smartphone overuse in daily life may simply be habitual use that does not satisfy the criteria of PSU, it may nonetheless occur automatically in the presence of smartphone-related cues when there is little conscious effort being made self-control.

Previous studies state an involvement of the ventral striatum in the experience of craving when being confronted with related cues were observed in subjects with Internet-gaming disorder (Ahn et al., 2015; Liu et al., 2017), hypersexual behavior (Klucken et al., 2016), and Internet-pornography use problems (Brand et al., 2016). Ventral striatum is the reward center of the brain- an important neuro-basis of conditioning. It also suggests that cue-induced behavior including craving play an important role in excessive smartphone use, whether it is defined as PSU or not.

Based on the aforementioned features of habitual behavior, then, it seems reasonable to speculate that the phone related cues might lead to automatic smart phone use on a daily basis such as unlocking the smartphone to check for notifications (Oulasvirta et al., 2012). When people are under the stimulation of phone cues, they might then increase the average frequencies and extend the durations of their smartphone. So, although smartphone overuse in daily life may simply be cue induced behavior that does not satisfy the criteria of PSU, it is just a kind of habitual behavior. It may nonetheless occur automatically in the presence of smartphone-related cues when there is no conscious effort being made self-control.

Smartphone overuse does not necessarily equate PSU then– it may merely enhance PSU (Van Deursen et al., 2015), and, as simply habitual behavior, it may be less harmful overall than PSU. However, it is important to be aware of the unnecessary stress that such habitual behavior may generate if people’s habitual phone use is arbitrarily labeled as being PSU. Therefore, it is essential to maintain a framework in which the two types of smartphone usage can be distinguished, to allow people to adjust their phone use accordingly. Such a framework would thus help those overusing their smartphones to understand the cue-induced mechanism behind their behavior, and adopt simple methods to decrease their phone use.

We conducted two experiments to test whether frequent use of smart phones can be affected through cued responses, and whether those responses would decline once the relevant cues are removed or as the individual becomes aware of their habitual use.

We hypothesized the following:

There will be more cue-induced smartphone use behavior in the group exposed the phone cues, which is reflected in their frequency and duration of smartphone use.

Awareness of phone use and attempts to control it can decrease the frequency and the duration of smartphone use.

## 2. Methods

### 2.1. Participants

The study sample comprised 62 college students (32 females and 30 males; mean age of 20.36 ± 1.68). They were randomly assigned to either the relevant cue group or the cue isolated group (1, 1). Two students completed the experiment quicker than expected and their results were therefore not included in the analysis. 34 students accepted the invitation to take part in the second experiment (17 females and 17 males, mean age of 20.27 ± 1.69). One participant who left early and three participants who used smart phone throughout the course, their data were excluded.

### 2.2. Study design

The study consist of two experiments. In the first experiment, Participants were randomly assigned to either the relevant cue group or the cue isolated group, and both groups completed the task under uncontrolled conditions. After 7 days, the second experiment were conducted, which is A 2 (control vs. no control) × 2 (cues vs. no cues) design. All participants in the first experiment were invited to be involved in the second one, which was conducted under self-controlled and no control two conditions with cue group and the cue isolated group.

### 2.3. Procedure

The experiment took place in the university library study room with a high-resolution indoor IP dome camera. All participants were told that they were taking part in a comfort survey in the library study room, where they were about to study with the camera on for 1 h and report on their experienced levels of comfort at the end of the hour; their smartphones needed to be set to silent mode to avoid disturbing others; there should be no conversation or discussion; and computers, MP3 players, and other electronic devices were not allowed. Participants’ behavior was recorded on camera for later analysis, and all of them were told that there are cameras in the library to record their behaviors. There were 17 trials in the experiment in total, with 5–7 participants from the same group (all from the cue group or all from the cue isolated group) assessed in each trial. Two types of behavior were coded and used as the dependent measures: Behavior 1, unlocking the smartphone but not sliding the screen, then turning off the screen quickly; and Behavior 2, unlocking the smartphone and slide screen, then continuing to use the phone.

Two postgraduates were trained in coding. In the training sessions, the coder coded the video data independently. The results were compared with the codes given by the more skilled coder (i.e., researcher) to examine the coding consistency. The kappa coefficient was used to evaluate inter-rater reliability. The consistency of the two coders in this study was 0.795, which shows that the coding results of the two coders had excellent consistency (Fleiss, 1981). Both coders were kept blinded during the allocation.

In the formal experiment, participants were randomly assigned to either the relevant cue group or the isolated cue group. In the relevant cue group, the participants were asked to place their smartphone on the desk, and the smartphone was always present in their visual field. In the cue isolated group, the participants were asked to place their smartphone in their bag or pocket, meaning that the smartphone was always absent in the visual field. There were no instructions given to either of the two groups to abstain from using the phone. Participants were asked to study while being recorded by the video camera for 1 h, but were given no directions as to whether they could use their smartphone or not. At the end of the hour they reported on their level of comfort during the session. The video content was coded for duration and frequency of smartphone use. The effect of the relevant cues was assessed by comparing the duration and frequency of smartphone use in the two groups.

Seven days later, those who had participated in the first experiment (in either of the relevant cues or no cues group) were invited to participate in the second experiment. The second experiment was similar to the first experiment, but this time the experimental group was asked to abstain from using their phone during the 1 h of videotaped study time. Before the session began, participants in the experimental condition were asked: “Could you try to be aware of your phone use behavior and attempt to not use your smart phone for the next hour?” The effect of asking the participants not to use their phone was assessed by comparing the duration and frequency of smartphone use in the two experiments.

### 2.4. Statistical analysis

A chi-square test and independent sample *t* test were used to measure and compare the frequency and the duration of smartphone use Behavior 1 and Behavior 2 between the experimental and control groups. Repeated measures ANOVA was then used to test the interactive effect of relevant cues and self-control on the duration of smartphone use.

## 3. Results

Participants in the relevant cue group displayed significantly more Behavior 1 than those in the cue isolated group (*χ*^2^ = 4.62, *df* = 1, *p* < 0.05), there was no significant group difference in frequencies of Behavior 2 (*χ*^2^ = 3.58, *df* = 1, *p* > 0.05). However, the duration of Behavior 2 was significantly longer in the relevant cue group than in the cue isolated group (*t* = 2.44, *p* < 0.05; see Table 1).

**Table 1:** Smartphone use frequencies and length for cue-induced group and no cues group (*N* = 60).

	Behavior 1		Behavior 2	
	Frequency	Length	Frequency	Length
Cue-induced group	18	39	44	9,003
No cues group	1	3	15	1,550

Both control intentions showed more Behavior 2 than Behavior 1. There was no significant difference between the two groups with regards to Behavior 1 (*χ*^2^ = 0.34, *df* = 1, *p* > 0.05). During the 1 h of observation, the occurrence rates for touching the phone (Behavior 1) were 81.1 and 79.2% in each group, respectively. However, the duration of Behavior 2 was significantly shorter in the self-control group than in the no self-control group (*t* = 2.44, *p* < 0.05; See Table 2).

**Table 2:** Smartphone use frequencies and length for conditions with will to control smart-phone use and without one for cues and cue isolated group (*N* = 30).

		Control condition		No control condition		Cue	Control	Cue×Control
		Frequency	Length	Frequency	Length	*F*	*F*	*F*
Behavior 1	Cue-induced group	5	17	7	25			
	No cues group	1	3	1	3			
Behavior 2	Cue-induced group	19	3,168	30	7,385	29.64*	5.68*	4.45*
	No cues group	3	462	12	1,150			

*p<0.05.

For behavior 2, the results showed a main effect for cues [*F* (1, 28) = 29.64, *p* < 0.05, *η*^2^ = 0.34] and intention of self-control [*F* (1, 28) = 5.678, *p* < 0.05, *η*^2^ = 0.09], with a significant interaction effect [*F* (1, 28) = 4.45, *p* < 0.05, *η*^2^ = 0.07]. For behavior 1, Simple effects analysis found that the self-control intention was significant only in the relevant cue [*F* (1, 28) = 10.01, *p* < 0.05], and not in the no-cue condition [*F* (1, 28) = 0.037, *p* > 0.05]. The relevant cue effect was significant in both the non-control intention group [*F* (1, 28) = 19.03, *p* < 0.05] as well as the self-control group [*F* (1, 28) = 9.68, *p* < 0.05]. For the behavior 1, there is no significant difference between two groups and two conditions.

## 4. Discussion

In this study, two behavioral experiments were conducted to explore whether cues (i.e., the presence or absence of a smartphone in participants’ visual field) would induce more frequent or longer periods of distraction. The results suggest the smartphone use can be induced by relevant cues, demonstrated by the fact that the relevant cue group exhibited more smartphone use behavior and used it for longer durations than the group without phone relevant cues. This may underlie daily smartphone overuse, in that a large part of smartphone use on a daily basis may be triggered by relevant cues, rather than being an example of PSU, and it is just habitual behavior learned through stimulus–response learning. The implication of these results is that excessive smartphone use could potentially be decreased by reducing phone-related cues.

Smartphone usage frequency is higher when the smartphone is in sight compared to when it is out of sight, even if people do not consciously intend to use it, suggesting that phone use is more likely to occur in response to the visual cue (i.e., seeing the phone) (Aarts and Dijksterhuis, 2000); response behaviors (i.e., using the smartphone) may then be automatic and occur unconsciously (Chou and Hsiao, 2000; Wood and Neal, 2007; Lopez-Fernandez et al., 2014).

This phenomenon is consistent with the characteristics of conditioned reflex behavior (Mulligan et al., 2000; Felisoni and Godoi, 2018). This model has been confirmed in research on problematic substance use (Zeng et al., 2018), for example, in the context of alcohol abuse, the stimulus of being in a bar is seen to evoke the response of drinking alcohol and using substances (LaRose and Eastin, 2004). Based on the results of the current study, this phenomenon may also inform smartphone use.

The effect of the relevant cues on frequency was not affected by the instruction to use deliberate self-control. This result was different than our expectation. It appears that regardless of whether an individual consciously intends to control their smartphone use, the presence of cues makes smartphone use (e.g., unlocking the smartphone) more likely to occur. This further suggests that smartphone use under relevant cues may be to a large extent unconscious, and therefore difficult to acknowledge and control. In this way, if relevant cues could be reduced, the frequency of smartphone usage might also be reduced.

However, our results also demonstrated that when participants entered a conscious state after being unaware of their actions, intentional self-control could reduce the duration of usage, meaning that self-control can be useful for reducing the phone use.

Furthermore, both groups displayed more of Behavior 2 (Unlocked and used smartphone) than Behavior 1 (Unlocked smartphone but did not use it), which suggests that once an individual touches their smartphone screen due to relevant cues, they are more likely to spend time on their smartphone and less likely to merely activate the screen before turning it back off, indicating that the content appearing on the phone screen may potentially play a role in cue-induced smartphone use and result in longer periods of smartphone use.

All the above results suggest that the use of phones in response to relevant cues may simply be conditioned habitual behavior, and is consistent with the characteristics of habitual behaviors in line with Oulasvirta who found that habitual behavior is automatic behavior triggered by situational cues (Oulasvirta et al., 2012). Our findings are also consistent with the results of LaRose and Eastin’s study (Whang et al., 2003), which established that habitual behavior is also behavioral acts that occur without conscious thought or self-instruction.

Overall, we believe that while the majority of smartphone overuse might be induced by cues, the behavior should not necessarily be classified as indicative of PSU. Smartphone use under the relevant cues is not so much PSU as habitual behavior. It is easy to impose psychological burden on people to be labeled PSU, particularly toward younger populations. Habitual smartphone use might be reduced by putting phone related cues out of sight or consciously paying attention to one’s smartphone use behavior in an effort to try to control it (i.e., reduce the duration of phone use). This would be useful in avoiding increasingly problematic behaviors which might even further lead to PSU. Huisman et al. have pointed out that Internet or other digital addictions are often an outcome of habitual behavior. If people try to reduce relevant cues at the stage of habitual behavior, this may help decrease the risk of PSU. Indeed, this is the focus of the current research, that simply reducing cues that trigger smartphone use may help people decrease their risk of developing PSU.

This study contributes to the growing body of literature on smartphone use by examining the role of visual cues and self-control in daily phone use, behavior. Furthermore, it indicates that much of daily smart-phone use may simply be habitual behavior spurred by relevant cues, rather than problematic smartphone use. By understanding the underlying factors contributing to excessive smartphone use, our findings can help inform more effective approaches for reducing the frequency and duration of smartphone use among college populations, ultimately promoting healthier digital habits.

### 4.1. Limitations

Smartphone use under relevant cues might be automatic behavior triggered by a variety of both external/situational and internal cues. The current study limited its focus to only a visual cue. There could be individuals whose habitual smartphone use has been formed through internal cues (e.g., boredom, loneliness, etc.), rendering the presence or absence of a visual cue irrelevant. The effect of internal cues on daily smartphone use should also be explored. In addition, we did not calculate the number of participants who did not use their smartphones at all under the relevant cues. This information might also help us better understand cue induced smartphone use.

## 5. Conclusion

This study investigated the role of visual cues and self-control in smartphone-related behavior among college students. Our findings revealed that the presence of smartphones in sight and the absence of instructions to exert self-control significantly increased cue-related phone use. These results suggest that visual cues and self-control may be important factors in driving excessive smartphone use, which may drive the daily smart phone use in the context of habitual behavior.

## Data availability statement

The original contributions presented in the study are included in the article/supplementary materials, further inquiries can be directed to the corresponding author/s.

## Ethics statement

The studies involving humans were approved by the Department of psychology, Guangzhou University, Guangzhou, China. The studies were conducted in accordance with the local legislation and institutional requirements. The participants provided their written informed consent to participate in this study. Written informed consent was obtained from the individual(s) for the publication of any potentially identifiable images or data included in this article.

## Author contributions

HZ made a substantial contribution to the concept and design of the work, also revised draft critically for important intellectual content. ML, JD, and JZ made a contribution to the acquisition, analysis, and interpretation of data. XY drafted the article and revised it critically. YL made a contribution to the data analysis and English language review. All authors have read and agreed to the published version of the manuscript.
